# Primary Care Prescribing Patterns for Benzodiazepines and Z-Drugs, and Evidence Gaps Concerning Hydroxyzine and Seasonality: A Systematic Review

**DOI:** 10.3390/clinpract16090166

**Published:** 2026-09-09

**Authors:** Małgorzata Romaszko, Anita Nowacka, Michał Majewski

**Affiliations:** 1 Department of Pharmacology and Toxicology, School of Medicine, Collegium Medicum, University of Warmia and Mazury, 10-726 Olsztyn, Poland; 2 Department of Pulmonology and Tuberculosis, Warmian-Mazurian Center of Pulmonary Disease, 10-357 Olsztyn, Poland

**Keywords:** primary care, insomnia, hypnotics, benzodiazepines, Z-drugs, prescribing patterns, seasonality

## Abstract

**Introduction:** Medications with sedative and hypnotic properties, such as benzodiazepines, the so-called ‘Z-drugs’, and hydroxyzine, are used in primary care. However, data regarding their prescribing patterns, particularly in the context of seasonality, remain limited. **Aim:** This review aims to assess the current knowledge regarding primary care prescribing patterns for medications with potential sedative and hypnotic effects, with particular emphasis on benzodiazepines, ‘Z-drugs’, and hydroxyzine. **Material and Methods:** The systematic review was conducted following the PRISMA guidelines. The PubMed, Web of Science and Scopus databases were searched. Fourteen original English-language publications from 2015 to 2026, which focused on the primary care prescription of selected groups of medications with hypnotic properties, were included. **Results:** Most of the studies included concerned benzodiazepines, with inconsistent prescribing patterns. Some studies reported a decrease in the rate of their prescription, whilst others indicated an increase, particularly among older people and patients with multimorbidity. Data concerning ‘Z-drugs’ were less extensive and showed heterogeneous prescribing trends across countries and study periods. Overall, the findings indicate substantial international variability in hypnotic prescribing in primary care. Evidence regarding hydroxyzine prescribing was particularly limited, with only one study specifically examining its prescribing patterns in primary care. Data on the seasonal prescribing patterns for all groups of medications were limited. **Conclusions:** Prescription of medications with sedative and hypnotic properties remains an important part of primary healthcare practice. Yet, significant research gaps remain regarding hydroxyzine prescribing and the seasonal variation in hypnotic and sedative prescribing.

## 1. Introduction

Hypnotics constitute a diverse group of pharmacological substances used to induce or improve the quality of sleep. An unequivocal categorisation of particular chemical substances as belonging to this group may be difficult, as the boundaries between sedatives, anxiolytics, and hypnotics are often blurred and overlap.

In the specialist literature, attempts have been made to classify medications used in the treatment of insomnia into three main groups. Hypnotics constitute the first group, which includes, amongst others, benzodiazepines, nonbenzodiazepine benzodiazepine receptor agonists (e.g., cyclopyrrolones), and melatonin receptor agonists. The second group consists of psychotropic medications with sedative effects, including antidepressants and antipsychotics. The third group comprises other substances used in clinical practice as sleep-inducing agents, such as antihistamines and orexin receptor antagonists.

Mental health disorders represent a substantial public health challenge worldwide and contribute considerably to the workload of primary care services [1,2]. Primary care physicians frequently provide care for patients with psychiatric disorders, including depression, anxiety disorders, and sleep disorders [3,4]. Growing numbers of patients seek consultations with their general practitioners (GPs) owing to symptoms of depression, anxiety disorders or sleep disorders [5,6]. Data from the literature suggest that up to 50% of primary care patients may experience sleep difficulties, including insomnia, excessive daytime sleepiness or sleep-related breathing disorders [7,8]. Furthermore, sleep disorders may co-occur with other chronic conditions treated in the primary care setting [9,10]. In such cases, GPs often decide to prescribe psychiatric medications or introduce symptomatic treatment for sleep disorders, making the prescription of hypnotics a key part of everyday clinical practice in primary care [11,12,13].

Psychiatric disorders, including sleep disorders, are characterised by seasonality, as has been already demonstrated in scientific studies [14,15,16]. For example, manic episodes peak during the summer months, which are distinguished by long daylight hours, whereas their frequency is lowest in winter, when days are shorter [17]. These findings suggest that seasonal factors may influence clinical presentation and treatment needs [12]. This phenomenon is also reflected in sleep disorders, particularly during periods of limited exposure to daylight [18,19]. The conducted research to date additionally suggests that the transition to daylight saving time, which is commonly practised in many countries, may have a detrimental effect on the internal biological clock and contribute to sleep disorders [20,21,22]. The natural light–dark cycle is the key mechanism for synchronising the circadian rhythm. Sudden shifts in time related to the transition to daylight saving time (summer time) or standard time (winter time) lead to a temporary desynchronisation between the endogenous biological clock and the environmental circadian rhythm, which may exacerbate symptoms of insomnia and potentially increase the need for pharmacological sleep promotion [23,24,25,26].

Although several studies have investigated the prescribing of selected hypnotics in primary care, the available evidence remains fragmented and has not yet been comprehensively synthesized. Furthermore, little is known about seasonal variations in the prescribing of these medications, and evidence regarding the use of hydroxyzine for the treatment of insomnia in adult primary care patients remains limited. A comprehensive review of prescribing patterns is therefore needed to evaluate current clinical practice, identify gaps in the available evidence, and assess the extent to which prescribing patterns are consistent with current evidence-based recommendations. This review sought to answer the following research question: What is the current evidence regarding prescribing patterns and seasonal variation in the prescribing of benzodiazepines and Z-drugs in primary care, and what evidence gaps exist regarding hydroxyzine?

## 2. Aim

This study aimed to analyse the current state of knowledge regarding prescribing patterns of benzodiazepines and Z-drugs in primary care, with particular attention to seasonal variations and evidence gaps concerning hydroxyzine.

## 3. Material and Methods

This systematic literature review was conducted following the PRISMA guidelines (Preferred Reporting Items for Systematic Reviews and Meta-Analyses) [27]. The completed PRISMA 2020 checklist is provided in Supplementary Table S4.

The methodological quality of the included studies was independently assessed by two reviewers using the Mixed Methods Appraisal Tool (MMAT), version 2018 [28]. Any disagreements were resolved through discussion until consensus was reached. Each study was evaluated according to the MMAT criteria appropriate for its study design. The quality assessment was performed after study selection, and the results are presented in Supplementary Table S1. A review protocol was developed a priori to guide the review process. However, the protocol was not prospectively registered in PROSPERO or any other publicly accessible registry.

The review focused on sleep-inducing medications belonging to the following groups:

• Benzodiazepines;
• Nonbenzodiazepines (the so-called ‘Z-drugs’, including cyclopyrrolone and imidazopyridine derivatives, e.g., zolpidem, zopiclone, zaleplon);
• Antihistamines (hydroxyzine).

Melatonin receptor agonists were excluded because their use cannot be comprehensively captured through prescription-based data sources. Orexin receptor antagonists were excluded due to their relatively recent introduction and limited adoption in routine clinical practice during the period covered by this review.

A comprehensive literature search was conducted in three electronic databases: PubMed, Scopus, and Web of Science. The search was designed to identify original research studies investigating prescribing patterns of hypnotic and/or sedative medications in the primary care setting.

Eligibility was restricted to English-language publications published between 2015 and 2026. Records retrieved outside this publication period were excluded during screening. Only studies conducted in humans were included. In PubMed, filters were applied for clinical studies, collected works, comparative studies, multicenter studies, observational studies, and validation studies. In Scopus, the search was restricted to articles published in English and involving human subjects. In Web of Science, searches were conducted in the title and abstract fields and restricted to relevant research publications.

The initial search strategy was based on a combination of terms referring to the primary care setting and prescribing, combined with medication-specific terms, including “zolpidem”, “zaleplon”, “zopiclone”, “lorazepam”, and “hydroxyzine”, using the Boolean operators AND and OR.

To further increase the sensitivity and comprehensiveness of the search, the databases were re-searched on 18 August 2026 using an expanded search strategy. The expanded strategy incorporated a broader range of terms referring to the primary care setting, prescribing, and hypnotic and sedative medications. Terms referring to the primary care setting, including “primary care”, “primary health care”, “primary healthcare”, “general practice”, “general practitioner”, “general practitioners”, “family practice”, “family physician”, “family physicians”, “family doctor”, and “family doctors”, were combined using the Boolean operator OR. These terms were combined using AND with prescribing-related terms, including “prescription” and “prescrib”, and with medication-specific terms, including “hypnotic”, “sedative”, “sleep medication”, “benzodiazepine”, “Z-drug”, “zolpidem”, “zopiclone”, “zaleplon”, “hydroxyzine”, “lorazepam”, “temazepam”, “nitrazepam”, “diazepam”, “alprazolam”, “clonazepam”, “estazolam”, “triazolam”, and “flunitrazepam”.

The complete database-specific search strategies used for the initial and updated searches are provided in Supplementary Table S2 and Table S3, respectively. The initial database searches, completed on 12 April 2026, identified 3290 records in total, comprising 552 records from PubMed, 1924 from Scopus, and 814 from Web of Science. The additional database search conducted on 18 August 2026 identified 1690 records in total, comprising 124 records from PubMed, 726 from Scopus, and 840 from Web of Science.

All records retrieved in the initial search were imported into Rayyan for reference management, duplicate identification, and screening [29].

Following automated and manual identification of duplicates, 1859 duplicate records were removed, leaving 1431 unique records for title and abstract screening.

Eligible studies were required to be original research articles investigating the prescription of benzodiazepines, non-benzodiazepine hypnotics (Z-drugs), including zolpidem, zopiclone, and zaleplon, or hydroxyzine. Studies had to concern general primary care populations and assess seasonality or temporal trends in the prescribing of the medications of interest. Studies based on broad population-level primary care datasets that included both adults and younger individuals were eligible, whereas studies restricted exclusively to paediatric populations or narrowly selected clinical populations were excluded. For the purposes of this review, primary care was defined as care provided in general practice, family medicine, or equivalent first-contact primary healthcare settings. Studies were eligible if prescriptions were issued by primary care physicians or if prescribing attributable to primary care physicians could be clearly identified and analysed separately within a broader dataset. Registration of patients in a primary care database alone, without identifiable primary care prescribing data, was not considered sufficient for inclusion. Studies were eligible regardless of the specific clinical indication for prescribing; therefore, prescriptions were not required to be explicitly associated with a diagnosis of insomnia, provided that prescribing data for the medications of interest could be identified. Studies based on prescription, dispensing, or claims data were eligible, provided that they allowed the prescribing patterns of the medications of interest in primary care to be assessed. Studies examining multiple medication groups were eligible only when data concerning the medications of interest could be clearly identified and analysed separately. Eligible study designs included observational, cohort, cross-sectional, time-series, and interventional studies, provided that they met the remaining eligibility criteria.

During the first stage of the selection process, 1431 records were assessed based on their titles and abstracts. At this stage, publications were excluded when the available information indicated that they did not meet the predefined eligibility criteria, particularly with regard to the healthcare setting, a narrowly selected population, the medications of interest, or relevance to medication prescribing. Following title and abstract screening, 86 articles were considered potentially eligible and were retrieved for full-text assessment. The full texts were subsequently assessed against all predefined eligibility criteria. The reasons for full-text exclusion in the initial search are presented in Table 1.

Among the 45 studies classified as involving an ineligible population, the excluded studies included those restricted to narrowly selected populations, such as individuals at risk of or with substance dependence, veterans, patients with cancer, or patients with chronic pain, as well as studies restricted to specific age groups rather than broad primary care populations. Following full-text assessment, 5 studies met all eligibility criteria and were included in the initial synthesis.

For the additional search conducted on 18 August 2026, following automated and manual duplicate identification, 850 duplicate records were removed from the updated search results, leaving 840 records for title and abstract screening. These records were assessed based on their titles and abstracts. At this stage, publications were excluded when the available information indicated that they did not meet the predefined eligibility criteria, particularly with regard to a narrowly selected population, a population not restricted to primary care, an overly broad definition of hypnotic medications, duplicate records identified in the initial search or during the review process, lack of assessment of prescribing patterns, or lack of assessment of seasonality or prescribing trends.

Following title and abstract screening, 61 articles were considered potentially eligible and were retrieved for full-text assessment. The full texts were subsequently assessed against all predefined eligibility criteria. The reasons for full-text exclusion in the additional search are also presented in Table 1. Among the 12 studies classified as involving an ineligible or selected population, the excluded studies included those restricted to narrowly selected populations, such as patients with a diagnosis of insomnia, patients with autism spectrum disorder, patients who had not previously attended a psychiatric visit, individuals with substance dependence, or patients with back pain, as well as studies restricted to specific age groups rather than broad primary care populations. Following full-text assessment, 9 additional studies met all eligibility criteria and were included in the qualitative synthesis.

The initial search identified 5 eligible studies, while the additional search conducted on 18 August 2026 identified 9 further eligible studies. Thus, a total of 14 studies were included in the systematic review.

Data extraction was performed by two reviewers using a structured framework covering study characteristics, population, medications analysed, prescribing-related outcomes, and main findings. Any discrepancies were resolved through discussion until consensus was reached. Studies based on overlapping datasets were considered separately only when they provided distinct analyses relevant to the review question. Owing to substantial heterogeneity in study populations, designs, healthcare settings, and prescribing measures, meta-analysis was considered inappropriate and the findings were synthesized narratively. The MMAT assessment was considered when interpreting the strength and generalizability of the evidence.

**Figure 1:**
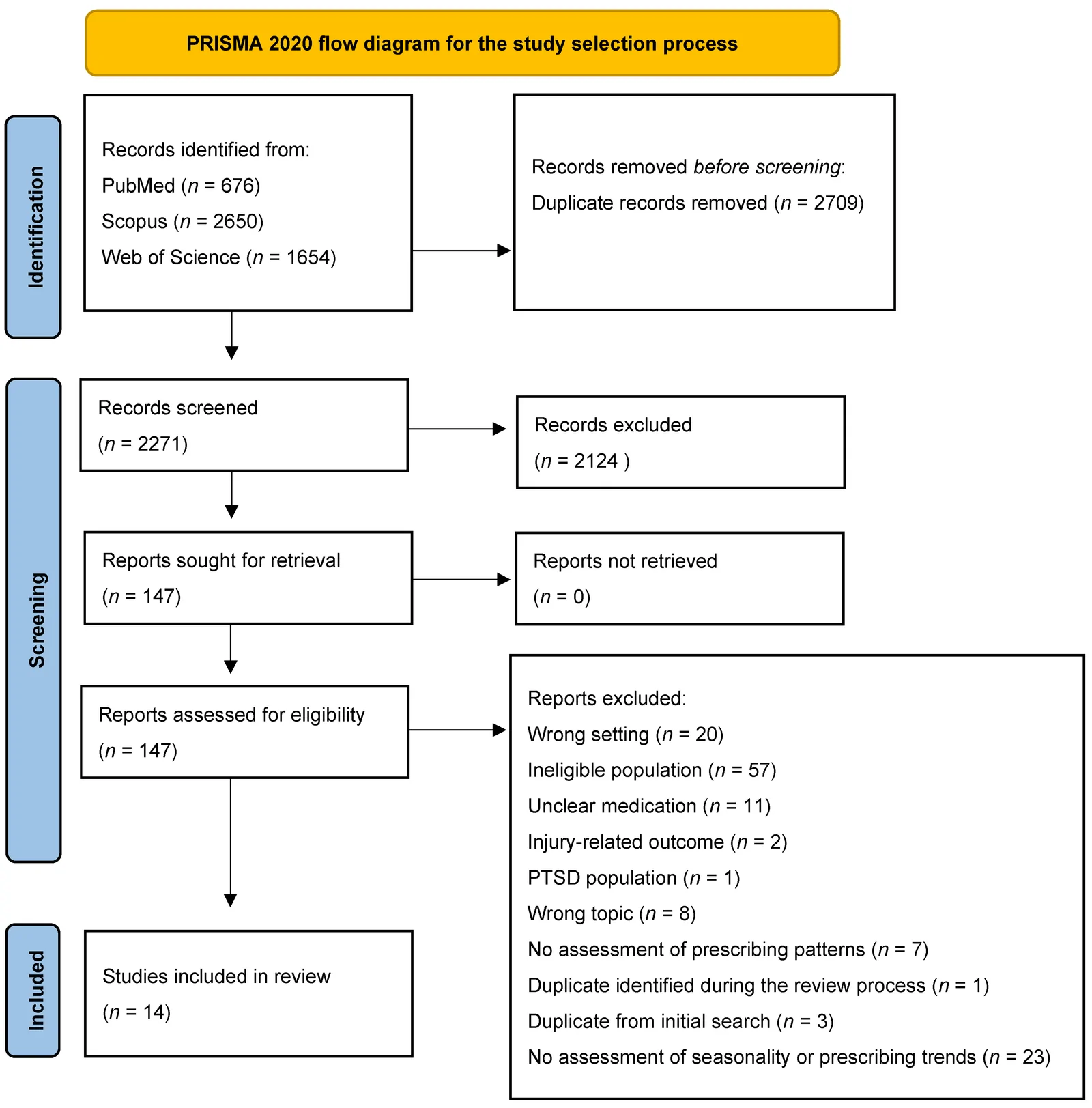
PRISMA 2020 flow diagram of the study selection process.

Additional relevant publications identified during the preparation of the manuscript, but outside the predefined systematic search and screening process, were used solely to provide contextual information and support the interpretation of the findings in the Discussion and were not considered part of the systematic review. The study selection process is presented in Figure 1.

## 4. Results

The review included 14 publications (Table 2) published between 2015 and 2026, providing data from various regions of the world, including Europe (10 publications—no. 1, 3, 4, 5, 7, 9, 11, 12, 13, and 14 in Table 2), the United States (2 publications—no. 2 and 10 in Table 2), and Australia (2 publications—no. 6 and 8 in Table 2). Among the included publications concerning hypnotics, most studies focused on benzodiazepines (13 publications—no. 1–13 in Table 2), while nonbenzodiazepine hypnotics, known as ‘Z-drugs’, including zolpidem, zopiclone, and zaleplon, were investigated in 8 publications (no. 2, 3, 6, 8, 9, 11, 12 and 13 in Table 2). Hydroxyzine was analysed in one publication (no. 14 in Table 2).

The majority of identified publications concerned benzodiazepines. These medications were the most frequently investigated group among the included studies, with several publications examining their prescribing in primary care settings (Table 2, no. 1–13). Long-term prescribing was also examined in some of the included studies.

Nonbenzodiazepine hypnotics, commonly referred to as ‘Z-drugs’, were investigated in several studies, frequently alongside benzodiazepines (Table 2, no. 2, 3, 6, 8, 9, 11, 12, and 13). Overall, the included studies demonstrated substantial variability in the medications investigated and the aspects of prescribing assessed across countries and healthcare systems.

The MMAT assessment indicated generally good methodological quality across the included studies. All studies met both screening criteria, while some methodological limitations were identified in individual studies, particularly regarding sample representativeness. Additional limitations were identified in selected non-randomized studies. Detailed results of the methodological quality assessment are presented in Supplementary Table S1.

Additionally, several studies analysed the impact of external factors on the prescribing of hypnotic and sedative medications. In particular, studies conducted during the COVID-19 pandemic examined changes in prescribing patterns in primary care (Table 2, no. 4, 5, 10, and 11). Another study examined prescribing following a community intervention aimed at reducing the use of potentially addictive medications (Table 2, no. 9).

The analysed publications provided limited evidence regarding the seasonality of hypnotic and sedative prescribing. One study specifically assessed seasonal variation in the prescribing of benzodiazepines and Z-drugs in primary care (Table 2, no. 13). The additional search conducted on 18 August 2026 identified a publication specifically examining seasonal and daylight saving time-related variation in hydroxyzine prescribing in primary care (Table 2, no. 14). This study reported significant seasonal variation in hydroxyzine prescribing, with the highest prescribing rates observed in winter and early spring and the lowest rates during summer. Prescribing was also inversely associated with day length.

## 5. Discussion

This systematic review demonstrates substantial international variability in prescribing hypnotics in primary care. Although benzodiazepines remain the most extensively studied medication group in this review, prescribing practices differ considerably between countries and patient populations. Furthermore, evidence on hydroxyzine prescribing in primary care remains particularly limited despite its off-label use as a sleep aid, and data on seasonal prescribing remain scarce.

### 5.1. Prescription Patterns and Trends

Benzodiazepines

Despite current guideline recommendations discouraging the long-term use of benzodiazepines, these medications continue to be commonly prescribed in primary care. Studies focusing on benzodiazepine prescribing predominated among the selected studies. Their findings were inconsistent: some studies reported a decline in benzodiazepine prescribing, whereas others showed an increase. More consistent findings concerned the increased prescribing rate of benzodiazepines in older patients and in those with comorbidities.

Although long-term benzodiazepine use remains common in a proportion of primary care patients, several studies have reported changes in prescribing patterns over time, including during the COVID-19 pandemic [33,36,41,44]. Weleff et al. (Table 2, no. 10) found that benzodiazepines were prescribed during 3.2% of primary care visits, with anxiety disorders showing the strongest positive association with prescribing. Contrary to their hypothesis, the odds of receiving a benzodiazepine prescription were 8.8% lower in the post-lockdown [39]. Other included studies conducted during the COVID-19 pandemic also demonstrated changes in prescribing patterns. Frazer et al. (Table 2, no. 5) found no significant changes for most benzodiazepines compared with predicted prescribing trends during the COVID-19 pandemic, although lorazepam prescribing increased by 10.2% in April 2020, while Kurvits et al. (Table 2, no. 11) observed a temporary increase in benzodiazepine and Z-drug use in Estonia following the onset of the pandemic [34,40].

Temporal trends varied considerably across countries and according to the type of benzodiazepine analysed. In Swedish primary care, Maun et al. (Table 2, no. 1) reported that benzodiazepine prescribing increased between 2011 and 2013 in both public and private practices, although prescribing levels were lower in private practices [30]. In Scotland, Weatherburn et al. (Table 2, no. 3) analysed benzodiazepine and Z-drug prescribing in primary care between 2015/16 and 2017/18. Although prescribing declined across all GP practice clusters, interrupted time-series analysis indicated that this reduction largely reflected an ongoing downward trend, with no significant additional effect attributable to the intervention. Further Australian data illustrate the complexity of benzodiazepine prescribing patterns [32]. Woods et al. (Table 2, no. 6) reported that the prevalence of long-term benzodiazepine and Z-drug prescribing increased from 4.4% in 2011 to 5.8% in 2015 and remained relatively stable thereafter until 2018 [35]. In a separate analysis of Australian general practice data covering the same period, Gonzalez-Chica et al. (Table 2, no. 8) found that prescribing of short- to intermediate-acting benzodiazepines decreased by 5.1% annually, while prescribing of long-acting benzodiazepines decreased by 1.5% annually [37]. In contrast, prescribing of very short-acting benzodiazepines increased by 17.2% annually. Z-drug prescribing decreased by 1.9% annually. In Irish primary care, Mattsson et al. (Table 2, no. 12) reported that dispensing of benzodiazepines and Z-drugs decreased by 5% between 2014 and 2022, while the use of alternative medications with sedative properties increased substantially [41].

Findings from studies that did not meet the eligibility for the present systematic review nevertheless provide additional context for benzodiazepine prescribing in primary care. An analysis by Moßhammer et al. covering the years 2009–2014 revealed that almost 5% of primary care patients in Germany received at least one prescription for benzodiazepines or nonbenzodiazepine hypnotic medications during one year of observation. The most frequently prescribed medication was lorazepam [45]. Mell et al., analysing German data from 2014, found that among older patients receiving benzodiazepines from GPs, sleep disorders were the most common psychiatric indication, recorded in 33.9% of patients. The proportion of patients receiving benzodiazepines increased with age, from 3.2% among those aged 65–69 years to 8.6% among those aged 90–100 years [46]. These findings confirm the widespread use of these medications in ambulatory settings.

Evidence from intervention studies suggests that long-term benzodiazepine and Z-drug use can be reduced in primary care [38]. In Norwegian primary care, Navaratnam et al. (Table 2, no. 9) reported that a multilevel intervention targeting GPs, patients, and the general public was associated with a 27% reduction in benzodiazepine prescribing and a 30% reduction in Z-drug prescribing between 2017 and 2020. These findings suggest that targeted interventions may effectively reduce the prescribing of these medications in primary care. A Finnish randomised controlled trial further demonstrated that gradual dose reduction combined with psychosocial support resulted in high short-term withdrawal rates among older primary care patients with long-term use of temazepam, zopiclone, or zolpidem; however, controlled-release melatonin provided no additional benefit over placebo [47]. Long-term follow-up showed that successful withdrawal persisted in a proportion of patients, with 28% remaining free of these medications after three years [48]. Overall, although benzodiazepine prescribing patterns vary across countries and over time, their widespread and long-term use remains an important concern in primary care, particularly given the associated safety risks, while available evidence suggests that targeted deprescribing interventions may effectively reduce their use [38,47,48].

### 5.2. Z-Drugs

Z-drugs appear to have partially replaced benzodiazepines in several countries, although prescribing patterns remain heterogeneous and this shift has not been consistently observed across studies.

Kaufmann et al. (Table 2, no. 2), using data from the National Ambulatory Medical Care Survey in the United States, reported a significant increase in benzodiazepine and Z-drug prescribing between 1993 and 2010. At the same time, an evident shift away from benzodiazepines towards nonbenzodiazepine medications was observed in the treatment of sleep disorders, particularly among patients with insomnia [31].

Mattsson et al. (Table 2, no. 12) reported that Z-drug dispensing in Irish primary care generally increased until 2021, followed by a small decline in 2022. Over the entire 2014–2022 study period, dispensing of zopiclone and zolpidem increased by 6% and 7%, respectively [41]. Despite these increases in individual Z-drugs, the combined dispensing of benzodiazepines and Z-drugs decreased by 5% between 2014 and 2022. The increase in Z-drug prescribing observed in some settings may reflect a shift in clinical practice away from traditional benzodiazepines towards nonbenzodiazepine hypnotics, as previously reported by Kaufmann et al. (Table 2, no. 2) [31].

Evidence from other included studies further demonstrates that Z-drug prescribing trends vary across countries, healthcare systems, and study periods. In Scottish primary care, Weatherburn et al. (Table 2, no. 3) observed an overall decline in benzodiazepine and Z-drug prescribing between 2015/16 and 2017/18 [32]. In Australia, Woods et al. (Table 2, no. 6) reported an increase in the prevalence of long-term benzodiazepine and Z-drug prescribing from 4.4% in 2011 to 5.8% in 2015, followed by relative stability until 2018 [35]. In a separate analysis of Australian general practice data, Gonzalez-Chica et al. (Table 2, no. 8) reported an average annual decline of 1.9% in Z-drug prescribing [37]. Furthermore, the Norwegian intervention study by Navaratnam et al. (Table 2, no. 9) demonstrated a 30% reduction in Z-drug prescribing between 2017 and 2020 following a multilevel intervention [38].

Overall, Z-drugs remain an important component of hypnotic prescribing in primary care, but the available evidence does not indicate a uniform temporal trend in their use. Prescribing patterns varied considerably across countries and study periods, with increases observed in some settings and declining or stable prescribing in others. These differences may partly reflect variation in healthcare organization and prescribing policies, as well as the effects of targeted interventions and external events. Across the included studies, changes in prescribing were observed in association with regulatory or practice-related changes and interventions aimed at reducing benzodiazepine and Z-drug use, while the COVID-19 pandemic was associated with temporary changes in prescribing patterns in several healthcare systems. Differences between countries may therefore reflect not only variation in clinical practice, but also differences in healthcare organization, prescribing policies, and responses to external events. However, as the included studies were not designed to directly compare these determinants across healthcare systems, their contribution to the observed international variability should be interpreted cautiously.

### 5.3. Hydroxyzine

Seasonal variation in hypnotic prescribing is biologically plausible because daylight exposure influences circadian rhythms and melatonin secretion. However, evidence on seasonal prescribing patterns in primary care remains scarce, particularly for hydroxyzine, despite its off-label use in routine clinical practice. Romaszko et al. (Table 2, no. 14) demonstrated significant seasonal variation in hydroxyzine prescribing in Polish primary care, with higher prescription rates during winter and early spring and lower rates during the summer months [43]. Prescription rates were also inversely associated with day length, suggesting a potential role of photoperiod in seasonal prescribing patterns [43]. Mattsson et al. (Table 2, no. 12) reported a 570% increase in the dispensing of sedating antihistamines in Irish primary care between 2014 and 2022; however, the available data did not allow hydroxyzine to be identified as a specific active agent [41]. The lack of comparable studies identified in this review highlights a significant research gap regarding hydroxyzine prescribing in primary care, particularly in the context of insomnia. Further research is therefore needed to characterize hydroxyzine prescribing patterns and to determine whether the observed seasonal variation can be replicated in other primary care populations and healthcare settings.

### 5.4. Assessment of Seasonality

Evidence regarding seasonal prescribing patterns for hypnotics and sedatives in primary care remains insufficient to determine whether a consistent seasonal pattern exists. Romaszko et al. (Table 2, no. 13) demonstrated significant seasonal variation in benzodiazepine and Z-drug prescribing in Polish primary care, with the lowest prescription rates observed during the summer months, particularly in July, and an inverse association between prescription rates and day length [42].

The following studies did not meet the eligibility criteria of the systematic review and are presented solely as contextual evidence; their findings were not used to support the principal conclusions of the review. Cadogan et al. analysed monthly benzodiazepine and Z-drug prescription claims in Ireland between 2016 and 2019. Their data showed short-term fluctuations in prescribing across individual months; however, these fluctuations did not demonstrate a consistent seasonal pattern and seasonality was not formally assessed [49]. Therefore, these findings provide only limited indirect evidence regarding potential seasonal variation in hypnotic prescribing.

Additional contextual evidence suggests that environmental light exposure may also be associated with hypnotic use. Min et al. (2018), in a population-based cohort of older adults in South Korea, found that greater exposure to outdoor artificial nighttime light was associated with more frequent hypnotic use [50]. Although this study did not meet the eligibility criteria of the present review and did not assess seasonal variation, its findings provide broader context regarding the potential relationship between environmental light exposure and hypnotic use.

Additional evidence suggests that monthly sunshine exposure may be associated with benzodiazepine use. Marković et al. (2021) found no significant differences in overall benzodiazepine consumption between spring–summer and autumn–winter periods; however, bromazepam consumption was negatively associated with monthly sunshine hours, with higher consumption observed during months with ≤131.45 h of sunshine [51]. These findings suggest that photoperiod-related environmental factors may be relevant to medication use even in the absence of a clear seasonal pattern.

The strength of the available evidence differed across the main findings of the review. Evidence regarding benzodiazepine prescribing was supported by the largest number of included studies, although temporal trends varied across countries and healthcare systems. Evidence regarding Z-drug prescribing was based on fewer studies and showed considerable heterogeneity. In contrast, evidence regarding hydroxyzine prescribing was particularly limited, with only one eligible study specifically examining hydroxyzine, while seasonal variation in prescribing was assessed in only a small number of eligible studies. These findings should therefore be interpreted cautiously until replicated in other populations and healthcare settings.

## 6. Limitations

This review has several limitations. First, evidence regarding seasonal variation in hypnotic prescribing was scarce, as most studies did not report prescribing patterns by season or month. Moreover, although all included studies concerned prescribing in primary care, the extent to which prescriptions could be specifically attributed to individual primary care physicians varied across studies. These limitations restrict the ability to assess potential seasonal factors influencing hypnotic and sedative prescribing specifically in primary care.

Second, most included studies assessed overall benzodiazepine and Z-drug prescribing without consistently distinguishing the clinical indication. Consequently, the extent to which the observed prescribing patterns specifically reflect the treatment of insomnia cannot be determined. Evidence regarding hydroxyzine prescribing in adult primary care was particularly limited, with only one eligible study identified, highlighting an important gap in the current literature.

Third, considerable heterogeneity existed among the included studies in terms of study populations, healthcare systems, observation periods, and measures of prescribing or dispensing, limiting direct comparisons between studies. Although all studies met both MMAT screening criteria, methodological limitations were identified in several studies, most commonly regarding sample representativeness among quantitative descriptive studies. These limitations should be considered when interpreting the generalizability of the findings.

Furthermore, the review was restricted to English-language publications, which may have introduced language bias and resulted in the omission of relevant studies published in other languages. In addition, the search was limited to PubMed, Scopus, and Web of Science, and relevant studies indexed elsewhere or not captured by the search strategy may have been missed. Therefore, the limited evidence identified, particularly regarding hydroxyzine and seasonal prescribing patterns, may partly reflect the database coverage and search approach used. The review protocol was developed a priori but was not prospectively registered in PROSPERO or another publicly accessible registry, which should also be acknowledged as a methodological limitation. Finally, publication bias cannot be excluded, as the review was based on published literature.

## 7. Conclusions

This systematic literature review highlights significant research gaps regarding hydroxyzine, a medication commonly used in clinical practice, including off-label, as a sleep aid. Although recent evidence from primary care has demonstrated seasonal variation in hydroxyzine prescribing and an association with daylight exposure, data remain scarce and require confirmation in other populations and healthcare settings. Evidence regarding Z-drug prescribing was more extensive but showed considerable heterogeneity, with increasing, decreasing, or stable prescribing patterns reported across different countries and study periods. Benzodiazepines were the most extensively studied medication group, although their prescribing patterns and temporal trends also varied across healthcare systems. Overall, the available evidence demonstrates substantial international variability in the prescribing of benzodiazepines and Z-drugs in primary care. Evidence regarding seasonal variation remains insufficient to determine whether consistent seasonal prescribing patterns exist across primary care populations. Although individual studies suggest possible associations with seasonality and daylight exposure, these findings remain preliminary and require confirmation in other populations and healthcare settings. Further research is therefore warranted to better characterize temporal and seasonal prescribing patterns, particularly given the limited evidence regarding hydroxyzine, and to identify factors contributing to variation in prescribing practices.

## Figures and Tables

**Table 1:** Reasons for full-text exclusion in the initial and additional literature searches.

Reason for Exclusion	Initial Search	Additional Search
Population not restricted to primary care	18	2
Selected population	45	12
Study concerned injuries associated with medication use	2	0
Population consisted of patients with PTSD	1	0
Wrong topic	8	0
No assessment of prescribing patterns	3	4
Specific medications could not be clearly identified	2	9
Duplicate identified during the review process	0	1
Duplicate of studies identified in the initial search	0	3
No assessment of seasonality or prescribing trends	2	21
Total	81	52

**Table 2:** Characteristics and main findings of studies included in the systematic review.

No	Authors	Title	Journal	Year	Country	Study Design/Data Source/Study Period	Population/Sample Size	Analysed Medications	Prescriber Definition	Prescribing Measure/Denominator	Main Findings/Numerical Estimates	Principal Limitations/MMAT Assessment
1	Andy Maun et al. [30]	Is the quality of primary healthcare services influenced by the healthcare centre’s type of ownership?—An observational study of patient perceived quality, prescription rates and follow-up routines in privately and publicly owned primary care centres	BMC Health Services Research	2015	Sweden	Retrospective observational study/regional healthcare authority data on purchased prescriptions/April 2011–January 2014	Primary care patients; 200 primary care centres (86 privately owned and 114 publicly owned)	Benzodiazepines	Prescriptions issued at primary care centres	Benzodiazepine DDD per 100 PCC patients/month	BZD use increased by 3.6% (age 20–74) and 7.4% (>74); lower in private PCCs.	Confounding not adequately accounted for; MMAT: C4 not met.
2	Kaufmann C. N. et al. [31]	Trends in prescribing of sedative-hypnotic medications in the United States: 1993–2010	Pharmacoepidemiology Drug Safety	2016	USA	Repeated cross-sectional trend analysis/NAMCS/1993–2010	516,118 patient visits	Benzodiazepines, Z-drugs	PCPs identifiable by specialty	% of ambulatory visits with BZD/nBZRA prescription	BZD: 2.6% → 4.4%; nBZRA: 0 → 1.4%; sleep-disorder visits: BZD 23.5% → 10.8%, nBZRA 2.3% → 13.7%	MMAT: all criteria met
3	Christopher J Weatherburn [32]	Benzodiazepines and non-benzodiazepine hypnotics—impact of a cluster adopted protocol on primary care prescribing	Scottish Medical Journal	2019	UK	Interrupted time series/NHS Scotland prescribing data/2015–2018	25 general practices (4 clusters)	Benzodiazepines and Z-drugs	GP practices	DDD per 1000 registered patients	Protocol effect: −0.4% (95% CI −7.2 to 7.6); NS.	MMAT: C1 not met
4	Carr M. J. et al. [33]	Effects of the COVID-19 pandemic on primary care-recorded mental illness and self-harm episodes in the UK: a population-based cohort study	The Lancet Public Health	2021	UK	Population-based cohort/CPRD EHR/2010–2020	14.2 million patients; 1697 GP practices	Benzodiazepines	GP prescriptions in CPRD	Prescription events per 100,000 person-months	BZD prescribing decreased sharply in April 2020, then recovered toward expected rates.	MMAT: all criteria met
5	Frazer J. S. et al. [34]	Analysis of primary care prescription trends in England during the COVID-19 pandemic compared against a predictive model	BMJ Journals Family Medicine and Community Health	2021	UK	Retrospective time-series/English Prescribing Dataset/2014–2020	7.54 billion prescriptions	Benzodiazepines	English primary care practices	Monthly prescriptions vs forecast	Lorazepam +10.2% (99% CI 1.9–19.9%) in Apr 2020; diazepam, clonazepam and temazepam unchanged.	Aggregate prescribing data; MMAT: all criteria met
6	Amelia Woods et al. [35]	long-term benzodiazepines and z-drug prescribing in Australian general practice between 2011 and 2018: A national study	Pharmacology Research & Perspectives	2022	Australia	Open cohort/MedicineInsight EHR/2011–2018	1,414,593 adults; 404 general practices	Benzodiazepines and Z-drugs	GP prescribing recorded in MedicineInsight	Long-term prescribing prevalence (≥3 prescriptions/6 months)	4.4% (2011) → 5.8% (2015); +2.5%/year (95% CI 2.0–3.0)	Limited sample representativeness; MMAT: C2 not met
7	Archer C. et al. [36]	Rise in prescribing for anxiety in UK primary care between 2003 and 2018: a population-based cohort study using Clinical Practice Research Datalink	British Journal of General Practice	2022	UK	Population-based cohort/CPRD GOLD/2003–2018	2,569,153 adults; 176 practices	Benzodiazepines	Prescriptions recorded in CPRD practices	Prevalence and incidence per 1000 PYAR	Incident BZD prescribing: 6.4 → 4.6/1000 PYAR	MMAT: all criteria met
8	David Gonzalez et al. [37]	Trends and patterns of benzodiazepines and Z-drugs prescriptions in Australian general practice: A national study (2011–2018)	Drug and Alcohol Review	2023	Australia	Open cohort/MedicineInsight EHR/2011–2018	1,450,613 adults; 50.8 million consultations; 402 practices	Benzodiazepines and Z-drugs	GP prescribing recorded in MedicineInsight	Prescriptions per 1000 consultations	Short/intermediate BZD: −5.1%/year; long-acting BZD: −1.5%/year; Z-drugs: −1.9%/year; very short-acting BZD: +17.2%/year	Limited sample representativeness; MMAT: C2 not met
9	Muhunthan Navaratnam et al. [38]	Prescription of potentially addictive medications after a multilevel community intervention in general practice	Scandinavian Journal of Primary Health Care	2023	Norway	Retrospective intervention study/Norwegian Prescription Registry/2017–2020	Prescription data from 26 of 36 GPs	Benzodiazepines and Z-drugs	GPs in Molde Municipality	DDD per 1000 listed patients	BZD −27%; Z-drugs −30% (2017–2020)	MMAT: C4 not met; C5 unclear
10	Weleff J et al. [39]	An Analysis of Benzodiazepine Prescribing to Primary Care Patients in a Large Healthcare System from 2019–2020	Journal of Psychoactive Drugs	2024	USA	Retrospective cohort/EHR/2019–2020	455,537 adults; 1,643,473 primary care visits	Benzodiazepines	Primary care providers	BZD prescriptions/primary care visits	BZD prescribed in 3.2% of visits; odds 8.8% lower post-lockdown	Limited sample representativeness; MMAT: C2 not met
11	Kurvits K. et al. [40]	The COVID-19 pandemic and the use of benzodiazepines and benzodiazepine-related drugs in Estonia: an interrupted time-series analysis	Child and Adolescent Psychiatry and Mental Health	2024	Estonia	Population-based ITS/Estonian Health Insurance Fund/2012–2021	397,436 individuals; 5,528,911 prescriptions	Benzodiazepines, Z-drugs	GPs identifiable by specialty	Monthly users per 1000 inhabitants	March 2020: +2.698/1000 (95% CI 1.408–3.988); no long-term overall change	MMAT: all criteria met
12	Molly Mattsson et al. [41]	Changes in benzodiazepine, z-drug, and other sedative prescribing in primary care in Ireland between 2014 and 2022	Family Practice	2025	Ireland	Repeated cross-sectional/GMS dispensing data/2014–2022	GMS population: 1.77 million (2014) → 1.57 million (2022)	Benzodiazepines, Z-drugs and antihistamines	Prescribed and dispensed in primary care under GMS	Dispensings per 1000 GMS population	BZD/Z-drugs −5%; BZD −13%; sedating antihistamines +570%	Limited sample representativeness; MMAT: C2 not met
13	Romaszko M. et al. [42]	Seasonal and gender-specific patterns in prescriptions for hypnotic and sedative medications in primary care	Frontiers in Psychiatry	2026	Poland	Retrospective study/EHR prescription data/2012–2024	716,242 adult consultations; ~13,000 registered patients	Benzodiazepines, Z-drugs	Primary care physicians	Daily prescription counts on working days	Lowest prescribing in May–Aug; significant reductions in Jun and Sep (*p* < 0.01) and Jul–Aug (*p* < 0.001)	Single-centre study; limited representativeness; MMAT: C2 not met
14	Romaszko M. et al. [43]	Seasonal and daylight saving time-related variation in hydroxyzine prescribing: Evidence of circadian and environmental modulation in primary care	Chronobiology International	2026	Poland	Retrospective study/EHR prescription data/2012–2024	13,625 adult consultations	Hydroxyzine	Primary care physicians	Daily prescriptions, adjusted for registered patients	Winter–summer increase: +11.4% women, +37.5% men; women 2.76× higher; no DST effect	Single-centre; limited representativeness; MMAT: C2 not met

Abbreviations: ATC, Anatomical Therapeutic Chemical classification; BZD, benzodiazepine; CI, confidence interval; CPRD, Clinical Practice Research Datalink; DDD, defined daily dose; DST, daylight saving time; EHR, electronic health record; GMS, General Medical Services; GP, general practitioner; ITS, interrupted time series; MMAT, Mixed Methods Appraisal Tool; NAMCS, National Ambulatory Medical Care Survey; nBZRA, nonbenzodiazepine benzodiazepine receptor agonist; NS, not significant; PCC, primary care centre; PCP, primary care physician; PYAR, person-years at risk.

## Data Availability

No new data were created or analyzed in this study.

## Supplementary Materials

The following supporting information can be downloaded at: https://www.mdpi.com/article/10.3390/clinpract16090166/s1, Table S1: MMAT assessment of the methodological quality of the included studies; Table S2: Initial search—12 April 2026; Table S3: Updated search—18 August 2026; Table S4: PRISMA 2020 Checklist.
