# Supplementary material for: Primary Care Prescribing Patterns for Benzodiazepines and Z-Drugs, and Evidence Gaps Concerning Hydroxyzine and Seasonality: A Systematic Review
Source: Clin Pract. 2026 Sep 9;16(9):166. doi: 10.3390/clinpract16090166 (PMC13605795; doi:10.3390/clinpract16090166)
Supplement: Supplementary file 1 [file clinpract-16-00166-s001.zip › Supplementary Tables.pdf]

Supplementary Table S1. MMAT assessment of the methodological quality of the included studies.

No.	Study	MMAT category	S1	S2	C1	C2	C3	C4	C5
1	Maun et al. (2015)	Quantitative non-randomized	Yes	Yes	Yes	Yes	Yes	No	Yes
2	Kaufmann et al. (2016)	Quantitative descriptive	Yes	Yes	Yes	Yes	Yes	Yes	Yes
3	Weatherburn (2019)	Quantitative non-randomized	Yes	Yes	No	Yes	Yes	Yes	Yes
4	Carr et al. (2021)	Quantitative non-randomized	Yes	Yes	Yes	Yes	Yes	Yes	Yes
5	Frazer & Frazer (2021)	Quantitative descriptive	Yes	Yes	Yes	Yes	Yes	Yes	Yes
6	Woods et al. (2022)	Quantitative descriptive	Yes	Yes	Yes	No	Yes	Yes	Yes
7	Archer et al. (2022)	Quantitative descriptive	Yes	Yes	Yes	Yes	Yes	Yes	Yes
8	Gonzalez-Chica et al. (2023)	Quantitative descriptive	Yes	Yes	Yes	No	Yes	Yes	Yes
9	Navaratnam et al. (2023)	Quantitative non-randomized	Yes	Yes	Yes	Yes	Yes	No	Can't tell
10	Weleff et al. (2024)	Quantitative descriptive	Yes	Yes	Yes	No	Yes	Yes	Yes
11	Kurvits et al. (2024)	Quantitative non-randomized	Yes	Yes	Yes	Yes	Yes	Yes	Yes
12	Mattsson et al. (2025)	Quantitative descriptive	Yes	Yes	Yes	No	Yes	Yes	Yes
13	Romaszko et al. (2026) – benzodiazepines/Z-drugs	Quantitative descriptive	Yes	Yes	Yes	No	Yes	Yes	Yes
14	Romaszko et al. (2026) – hydroxyzine	Quantitative descriptive	Yes	Yes	Yes	No	Yes	Yes	Yes

Supplementary Table S2. Initial search – 12 April 2026

Database	Search string	Records retrieved
Scopus	general practice OR primary care OR general practitioner AND prescription AND zolpidem OR zaleplon OR zopiclone OR hydroxyzine OR benzodiazepines OR lorazepam AND PUBYEAR > 2014 AND PUBYEAR < 2027 AND (LIMIT-TO (EXACTKEYWORD , "Human")) AND (LIMIT-TO (LANGUAGE , "English"))	1924
PubMed	((((((primary care[Title/Abstract]) AND (prescription[Title/Abstract])) AND (zolpidem[Title/Abstract])) OR (zaleplon[Title/Abstract])) OR (zopiclone[Title/Abstract])) OR (lorazepam[Title/Abstract]) OR (benzodiazepines[Title/Abstract])) OR (hydroxyzine[Title/Abstract])<br><br>((((((general practice[Title/Abstract]) AND (prescription[Title/Abstract])) AND (zolpidem[Title/Abstract])) OR (zaleplon[Title/Abstract])) OR (zopiclone[Title/Abstract])) OR (lorazepam[Title/Abstract])OR (benzodiazepines[Title/Abstract])) OR (hydroxyzine[Title/Abstract])<br><br>((((((general practitioner[Title/Abstract]) AND (prescription[Title/Abstract])) AND (zolpidem[Title/Abstract])) OR (zaleplon[Title/Abstract])) OR (zopiclone[Title/Abstract])) OR (lorazepam[Title/Abstract]) OR (benzodiazepines[Title/Abstract])) OR (hydroxyzine[Title/Abstract])	552
Web of Science	TI=((general practice AND prescription AND zolpidem) OR zaleplon OR zopiclone OR lorazepam OR hydroxyzine)<br><br>TI=((general practitioner AND prescription AND zolpidem) OR zaleplon OR zopiclone OR lorazepam OR hydroxyzine) OR AB=((general practice AND prescription AND zolpidem) OR zaleplon OR zopiclone OR lorazepam OR benzodiazepines OR hydroxyzine)<br><br>TI=((primary care AND prescription AND zolpidem) OR zaleplon OR zopiclone OR lorazepam OR hydroxyzine) OR AB=((general practice AND prescription AND zolpidem) OR zaleplon OR zopiclone OR lorazepam OR benzodiazepines OR hydroxyzine)	814

	AB=((general practice AND prescription AND zolpidem) OR zaleplon OR zopiclone OR lorazepam OR benzodiazepines OR hydroxyzine)<br><br>AB=((general practitioner AND prescription AND zolpidem) OR zaleplon OR zopiclone OR lorazepam OR benzodiazepines OR hydroxyzine)<br><br>AB=((primary care AND prescription AND zolpidem) OR zaleplon OR zopiclone OR lorazepam OR benzodiazepines OR hydroxyzine)	

Supplementary Table S3. Updated search – 18 August 2026

Database	Search string	Records retrieved
Scopus	TITLE-ABS ("primary care" OR "primary health care" OR "primary healthcare" OR "general practice" OR "general practitioner" OR "general practitioners" OR "family practice" OR "family physician" OR "family physicians" OR "family doctor" OR "family doctors") AND TITLE-ABS (prescription* OR prescrib*) AND TITLE-ABS (hypnotic* OR sedative* OR "sleep medication" OR "sleep medications" OR benzodiazepine* OR "Z-drug" OR "Z-drugs" OR zolpidem OR zopiclone OR zaleplon OR hydroxyzine OR lorazepam OR temazepam OR nitrazepam OR diazepam OR alprazolam OR clonazepam OR estazolam OR triazolam OR flunitrazepam) AND (LIMIT-TO (DOCTYPE , "ar")) AND (LIMIT-TO (LANGUAGE , "English")) AND (LIMIT-TO (EXACTKEYWORD , "Human"))	726
PubMed	("primary care"[Title/Abstract] OR "primary health care"[Title/Abstract] OR "primary healthcare"[Title/Abstract] OR "general practice"[Title/Abstract] OR "general practitioner"[Title/Abstract] OR "general practitioners"[Title/Abstract] OR "family practice"[Title/Abstract] OR "family physician"[Title/Abstract] OR "family physicians"[Title/Abstract] OR "family doctor"[Title/Abstract] OR "family doctors"[Title/Abstract]) AND (prescription*[Title/Abstract] OR prescrib*[Title/Abstract]) AND (hypnotic*[Title/Abstract] OR sedative*[Title/Abstract] OR "sleep medication"[Title/Abstract] OR "sleep medications"[Title/Abstract] OR benzodiazepine*[Title/Abstract] OR "Z-drug"[Title/Abstract] OR "Z-drugs"[Title/Abstract] OR zolpidem[Title/Abstract] OR zopiclone[Title/Abstract] OR zaleplon[Title/Abstract] OR	124

	hydroxyzine[Title/Abstract] OR lorazepam[Title/Abstract] OR temazepam[Title/Abstract] OR nitrazepam[Title/Abstract] OR diazepam[Title/Abstract] OR alprazolam[Title/Abstract] OR clonazepam[Title/Abstract] OR estazolam[Title/Abstract] OR triazolam[Title/Abstract] OR flunitrazepam[Title/Abstract]) AND (clinicalstudy[Filter] OR collectedwork[Filter] OR comparativestudy[Filter] OR multicenterstudy[Filter] OR observationalstudy[Filter] OR validationstudy[Filter]) AND humans[Filter] AND english[Filter] AND 2012:2026[pdat]	
Web of Science	("primary care" OR "primary health care" OR "primary healthcare" OR "general practice" OR "general practitioner" OR "general practitioners" OR "family practice" OR "family physician" OR "family physicians" OR "family doctor" OR "family doctors") AND (prescription* OR prescrib*) AND (hypnotic* OR sedative* OR "sleep medication" OR "sleep medications" OR benzodiazepine* OR "Z-drug" OR "Z-drugs" OR zolpidem OR zopiclone OR zaleplon OR hydroxyzine OR lorazepam OR temazepam OR nitrazepam OR diazepam OR alprazolam OR clonazepam OR estazolam OR triazolam OR flunitrazepam)<br>Fields searched: Title and Abstract	840
