# Supplementary material for: Primary Care Prescribing Patterns for Benzodiazepines and Z-Drugs, and Evidence Gaps Concerning Hydroxyzine and Seasonality: A Systematic Review
Source: Clin Pract. 2026 Sep 9;16(9):166. doi: 10.3390/clinpract16090166 (PMC13605795; doi:10.3390/clinpract16090166)
Supplement: Supplementary file 1 [file clinpract-16-00166-s001.zip › Table S4-PRISMA 2020 Checklist.pdf]

PRISMA 2020 Checklist

Primary Care Prescribing Patterns for Benzodiazepines and Z-Drugs, and Evidence Gaps Concerning Hydroxyzine and Seasonality: A Systematic Review

Section and topic	Item	Checklist item	Location where item is reported
TITLE	1	Identify the report as a systematic review.	Title, p. 1
ABSTRACT	2	See the PRISMA 2020 for Abstracts checklist.	Abstract, p. 1
INTRODUCTION – Rationale	3	Describe the rationale for the review in the context of existing knowledge.	Introduction, pp. 1–2
INTRODUCTION – Objectives	4	Provide an explicit statement of the objective(s) or question(s) the review addresses.	Introduction/Aim, pp. 2–3
METHODS – Eligibility criteria	5	Specify the inclusion and exclusion criteria for the review and how studies were grouped for the syntheses.	Materials and Methods, pp. 3–5
METHODS – Information sources	6	Specify all databases, registers, websites, organisations, reference lists and other sources searched or consulted to identify studies. Specify the date when each source was last searched or consulted.	Materials and Methods, pp. 3–4; Supplementary Tables S3 and S4
METHODS – Search strategy	7	Present the full search strategies for all databases, registers and websites, including any filters and limits used.	Supplementary Tables S3 and S4
METHODS – Selection process	8	Specify the methods used to decide whether a study met the inclusion criteria of the review, including how many reviewers screened each record and report retrieved, whether they worked independently, and if applicable, details of automation tools used.	Materials and Methods, pp. 4–5; Rayyan described on p. 4.
METHODS – Data collection process	9	Specify the methods used to collect data from reports, including how many reviewers collected data from each report, whether they worked independently, any processes for obtaining or confirming data from study investigators, and if applicable, details of automation tools used.	Materials and Methods, p. 5 (data extraction by two reviewers; discrepancies resolved by discussion)
METHODS – Data items	10a	List and define all outcomes for which data were sought. Specify whether all results compatible with each outcome domain in each study were sought, and if not, the methods used to decide which results to collect.	Materials and Methods, pp. 4–5; Table 2, pp. 8–10
METHODS – Data items	10b	List and define all other variables for which data were sought (e.g. participant and intervention characteristics, funding sources). Describe any assumptions made about any missing or unclear information.	Materials and Methods, p. 5 (study characteristics, population, medications, prescribing-related outcomes and main findings); Table 2, pp. 8–10
METHODS – Study risk of bias assessment	11	Specify the methods used to assess risk of bias in the included studies, including details of the tool(s) used, how many reviewers assessed each study and whether they worked independently, and if applicable, details of automation tools used.	Materials and Methods, p. 3; Supplementary Table S1 (MMAT 2018)
METHODS – Effect measures	12	Specify for each outcome the effect measure(s) used in the synthesis or presentation of results.	Table 2, pp. 8–10 (study-specific prescribing measures and numerical estimates); no pooled effect measure because meta-analysis was not performed
METHODS – Synthesis methods	13a	Describe the processes used to decide which studies were eligible for each synthesis.	Materials and Methods, pp. 4–5
METHODS – Synthesis methods	13b	Describe any methods required to prepare the data for presentation or synthesis, such as handling of missing summary statistics or data conversions.	Not applicable / no data conversion or statistical pooling performed

Section and topic	Item	Checklist item	Location where item is reported
METHODS – Synthesis methods	13c	Describe any methods used to tabulate or visually display results of individual studies and syntheses.	Table 2, pp. 8–10; narrative synthesis in Results and Discussion
METHODS – Synthesis methods	13d	Describe any methods used to synthesize results and provide a rationale for the choice(s). If meta-analysis was performed, describe the model(s), methods to identify statistical heterogeneity, and software package(s) used.	Materials and Methods, pp. 5–6: narrative synthesis; meta-analysis considered inappropriate because of substantial heterogeneity
METHODS – Synthesis methods	13e	Describe any methods used to explore possible causes of heterogeneity among study results.	No formal subgroup or meta-regression analysis was performed; potential sources of heterogeneity were discussed narratively in the Discussion, particularly on p. 14.
METHODS – Synthesis methods	13f	Describe any sensitivity analyses conducted to assess robustness of the synthesized results.	Not applicable; no meta-analysis/sensitivity analysis performed
METHODS – Reporting bias assessment	14	Describe any methods used to assess risk of bias due to missing results in a synthesis (arising from reporting biases).	No formal reporting-bias assessment; publication bias acknowledged as a limitation, p. 16
METHODS – Certainty assessment	15	Describe any methods used to assess certainty (or confidence) in the body of evidence for an outcome.	MMAT assessment considered when interpreting strength/generalizability, pp. 3 and 5–6; no separate formal certainty-grading framework used
RESULTS – Study selection	16a	Describe the results of the search and selection process, from the number of records identified in the search to the number of studies included in the review, ideally using a flow diagram.	Materials and Methods, pp. 4–6; Figure 1, p. 7
RESULTS – Study selection	16b	Cite studies that might appear to meet the inclusion criteria, but which were excluded, and explain why they were excluded.	Table 1, p. 5 (reasons for full-text exclusion); selected excluded studies used as contextual evidence are cited in the Discussion, pp. 12–15.
RESULTS – Study characteristics	17	Cite each included study and present its characteristics.	Table 2, pp. 8–10
RESULTS – Risk of bias in studies	18	Present assessments of risk of bias for each included study.	Table 2, pp. 8–10; Supplementary Table S1
RESULTS – Results of individual studies	19	For all outcomes, present, for each study: (a) summary statistics for each group (where appropriate) and (b) an effect estimate and its precision (e.g. confidence/credible interval), ideally using structured tables or plots.	Table 2, pp. 8–10; Results/Discussion, pp. 11–15
RESULTS – Results of syntheses	20a	For each synthesis, briefly summarise the characteristics and risk of bias among contributing studies.	Results, p. 11; Discussion, pp. 12–15; MMAT summary p. 11
RESULTS – Results of syntheses	20b	Present results of all statistical syntheses conducted. If meta-analysis was done, present for each the summary estimate and its precision and measures of statistical heterogeneity. If comparing groups, describe the direction of the effect.	Not applicable; no meta-analysis/statistical synthesis performed. Narrative synthesis, pp. 11–15
RESULTS – Results of syntheses	20c	Present results of all investigations of possible causes of heterogeneity among study results.	No formal heterogeneity investigation; possible explanations discussed narratively, pp. 13–14
RESULTS – Results of syntheses	20d	Present results of all sensitivity analyses conducted to assess the robustness of the synthesized results.	Not applicable; no sensitivity analyses performed
RESULTS – Reporting biases	21	Present assessments of risk of bias due to missing results (arising from reporting biases) for each synthesis assessed.	Not formally assessed; publication bias acknowledged in Limitations, p. 16

Section and topic	Item	Checklist item	Location where item is reported
RESULTS – Certainty of evidence	22	Present assessments of certainty (or confidence) in the body of evidence for each outcome assessed.	Narrative assessment of strength of evidence, p. 15; MMAT summary, p. 11; no formal GRADE-type assessment
DISCUSSION	23a	Provide a general interpretation of the results in the context of other evidence.	Discussion, pp. 11–15
DISCUSSION	23b	Discuss any limitations of the evidence included in the review.	Limitations, pp. 15–16
DISCUSSION	23c	Discuss any limitations of the review processes used.	Limitations, pp. 15–16
DISCUSSION	23d	Discuss implications of the results for practice, policy, and future research.	Discussion/Conclusions, pp. 13–16
OTHER INFORMATION – Registration and protocol	24a	Provide registration information for the review, including register name and registration number, or state that the review was not registered.	Materials and Methods, p. 3: protocol not prospectively registered
OTHER INFORMATION – Registration and protocol	24b	Indicate where the review protocol can be accessed, or state that a protocol was not prepared.	Materials and Methods, p. 3: a protocol was developed a priori but was not prospectively registered in PROSPERO or another publicly accessible registry.
OTHER INFORMATION – Registration and protocol	24c	Describe and explain any amendments to information provided at registration or in the protocol.	No amendments to a registered protocol were applicable because the review was not registered. The expanded updated search is described in Materials and Methods, pp. 3–4.
OTHER INFORMATION – Support	25	Describe sources of financial or non-financial support for the review, and the role of the funders or sponsors in the review.	Funding statement, p. 16: None declared
OTHER INFORMATION – Competing interests	26	Declare any competing interests of review authors.	Conflicts of Interest, p. 16: None declared
OTHER INFORMATION – Availability of data, code and other materials	27	Report which of the following are publicly available and where they can be found: template data collection forms; data extracted from included studies; data used for all analyses; analytic code; any other materials used in the review.	Data Availability Statement, p. 16. Supplementary materials include the MMAT assessment and complete database-specific search strategies (Supplementary Tables S1, S3 and S4).
